# The effect of Bosentan on healing of colonic anastomosis

**DOI:** 10.1186/1749-7922-1-37

**Published:** 2006-12-18

**Authors:** Ziya Cetinkaya, Kazim Esen, Ibrahim Hanefi Ozercan, Bilal Ustundag, Refik Ayten, Erhan Aygen

**Affiliations:** 1 Department of General Surgery, Faculty of Medicine, Firat University, 23100, Elazig, Turkey; 2 Department of Pathology, Faculty of Medicine, Firat University, 23100, Elazig, Turkey; 3 Department of Biochemistry, Faculty of Medicine, Firat University, 23100, Elazig, Turkey

## Abstract

**Background:**

Ischemia is the most important factor compromises wound healing in colonic anastomosis. Mesenteric vessels are ligated at first while performing colonic resection and following anastomosis. Therefore blood supply of the related segments of colon temporarily interrupted and ischemia can easily occur. This study was carried out to explore whether Bosentan, an endothelin-receptor antagonist, can eliminate vasoconstruction, increase blood flow in the splanchnic area and anastomotic region and therefore possibly facilitate wound healing and prevent intra-abdominal adhesion formation.

**Metods:**

Study is conducted on 30 female Wistar-Albino rats weighing 180–240 gr. Rats were allocated into three groups. Group 1 (n = 10) recevied full-thickness resection of the left colon and end-to-end anastomosis. In Groups 2 (n = 10) and 3 (n = 10), vessels of 2–3 cm segment of the left colon were ligated, indications of necrosis of that segment were expected, followed by resection and end-to-end anastomosis. Two milliliter of saline and 5 mg/kg Bosentan was given intraperitoneally in Group 2 and 3, respectively. On postoperativ day 6, intra-abdominal adhesions were scored. Healing of anastomosis, anastomotic bursting pressures, tissue hydroxyproline levels and histopatologically healing scores were assessed.

**Results:**

Macroscopic adhesion score in Group 3 was lower than the remained groups (p < 0.05). Tissue hydroxyproline levels were significantly higher in Group 3 compared to the Groups 1 and 2 (p < 0.001). Mean anastomotic bursting pressures were 200 mmHg, 164 mmHg and 240 mmHg in Groups 1, 2 an 3, respectively (p < 0.05 between Groups 1 and 3; p < 0.001 between Groups 2 and 3). Histopathologically, healing scores of Group 1 were significantly higher than the other groups (p < 0.05 group 1–3, group 2–3).

**Conclusion:**

Bosentan increases anastomotic healing of ischemic colonic anastomosis and decreases intra-abdominal adhesion formation.

## Background

Single-stage resection and anastomosis of lthe left colon has important advantages in reducing the hospitalization period, decreasing mortality and morbidity and protecting the patient from stoma. Anastomotic leak (15%), wound infection (50%) and intra-abdominal abscesses can be seen following colon resection (without colon cleansing) and primary anastomosis (RPA) of the left colon. There are numerous factors that disturb wound healing at the site of colon anastomosis. These include tension on the anastomosis, ischemia, distal obstruction, infection, surgical technique, malnutrition, certain drugs and disorders of collagen metabolism. Among these, ischemia is the most important factor [1-3]. The first step before the resection and anastomosis of colon is the ligation of mesenteric vessels. During this procedure the related segment of colon is exposed to ischemia for some time. Following the anastomosis ischemia can also occur on the suture line.

Drugs that inhibit vasoconstriction and increase blood flow may contribute to rapid wound healing on the anastomotic site. Endothelins are known the most potent vasoconstrictors. In addition to this effect, they play roles in water and electrolyte balance, regulation on the release of certain hormones, cell proliferation and vascular permeability [4,5]. Increased local endothelin has been shown to be the cause of increased vascular resistance in experimental strangulated small bowel obstruction model [6]. Three types of endothelin receptors, ET_A_, ET_B_, and ET_C _have been identified. Bosentan (Ro 47-0203) is a competitive antagonist of both ET_A _and ET_B_. It has been shown that Bosentan inhibited the increase in vascular resistance in strangulated small bowel [6]. Although the exact cause of intra-abdominal adhesion formation is unknown, ischemia is the most frequently implicated factor [7]. Recent English literature has not been focused on the effects of Bosentan on colonic wound healing and intra-abdominal adhesion formation.

The objective of this study was to determine the effect of Bosentan on ischemic colon anastomosis and intra-abdominal adhesion formation.

## Materials and methods

### Selection of rats

Upon an approval was obtained from the Ethics Board, this study was conducted to laboratories of Firat University Faculty of Medicine Experimental Research Center (FUTDAM), using 30 female Wistar-Albino rats weighing 180–240 g. Rats were fed standard pellet and tap water and were kept in room temperature and humidity controlled environment. Rats were randomly allocated to one of three groups containig 10 rats each.

Group 1 (G_1_): normal resection + anastomosis

Group 2 (G_2_): ischemia + resection + anastomosis

Group 3 (G_3_): ischemia + resection + anastomosis + Bosentan

### Operation procedure

Intramuscular 35 mg/kg Ketamin HCl (Ketalar, 50 mg/ml, Eczacibasi, Istanbul) and 5 mg/kg Xylazine HCl (Rompun, 23.32 mg/ml, Bayer, Istanbul) was injected to the right and left hind legs, respectively, to induce anesthesia in all groups. About 5 minutes prior to the operation the abdomen was shaved, cleaned with 10% Povidon iodine solution and draped with sterile drapes. Using sterile instruments, laparotomy was performed by midline incision. Group 1 received resection of the left colon 2.5 cm above the pelvic peritoneum and end-to-end anastomosis. Two milliliters of saline was administered in the peritoneal cavity. The abdomen was closed by 3/0 silk sutures. In Groups 2 and 3, mesenteric vessels of the 2–3 cm segment of the left colon 2.5 cm above the pelvic peritoneum was ligated with 4/0 silk suture thread. One hour following the procedure, at least one of the signs of pre-necrosis were evident, segment of the colon was transsected end-to-end anastomosis was carried out. In Group 2, 2 ml of saline and in Group 3, 5 mg/kg Bosentan was administered into the peritoneal cavity following the procedure. All groups end-to-end anastomosis was performed by full-thickness continuous sutures using 5/0 PDS. Rats were allowed water and standart laboratory feeding 24 hours following the operation. Rats were sacrificed on postoperative day 6.

Bosentan (Ro 470203) sodium was obtained to courtesy of Dr. Martine Clozel (F. Hoffman-La Roche Ltd, Basel, Switzerland). Twenty milligrams of Bosentan was dissolved in 20 ml distilled water and added to 20 ml saline to a total of 40 ml and diluated to obtain 1 mg Bosentan sodium in 2 ml. Dose selection was based on the results of previous studies [6,8]

### Assessment of adhesion formation

On postoperative day 6, all rats wer anesthesised, sutures were removed and the abdomen was opened to evaluate the healing of the anastomosis and adhesion formation. Intra-abdominal adhesions were assessed and graded by two surgeons blind to the groups of the rats using a standard scale according to the following criteria [9]. O point: No adhesion; 1 point: Single, easily dissectible adhesion; 2 points: Multiple, easily dissectible adhesion; 3 points: Single, dense adhesion; 4 points: Multiple, dense adhesions.

### Measuring the bursting pressure of the anastomosis site

Once the intra-abdominal adhesions were scored, without dissecting the adhesions, anastomotic bursting pressures were measured in-vivo using the setup described below by two surgeons blind to the groups.

A catheter is inserted through the anus and advanced 2–3 cm proximally so that the tip of the catheter lay at the level of the anastomosis. Peritoneal cavity was filled with saline. Colon was tied with 2/0 silk at 2 cm above and below the suture line. With both ends tied and a catheter inside, the segment of the colon was filled with saline colored with methylene blue at a rate of 4 ml/min using an infusion pump (Abbott LC 5000 infuser, USA). During the infusion, pressurs were monitored (Petas KMA 375 S/N 0013 Turkey) by a pressure transducer (Abbott Single Transpact, USA). The pressure value was taken while blue intracolonic flued leaked out to the peritoneal cavity and recorded as the bursting pressure.

### Obtaining the samples

A 2-cm segment of the colon including the anastomosis was resected, transsected longitudinally and rinsed with saline to remove intestinal contents. One third of this sample was fixed in 10% formalin for histopathological examination. Remaining two-thirds, wrapped in aluminum foil, was kept in biochemistry laboratory for tissue hydroxyproline measuring.

### Histopathological examination

Tissue samples obtained from the anastomotic site were embedded into paraffin blocks following routine histochemical procedures. Four-five micron thick sections were stained with Hematoxylin-Eosin and examined under light microscope. Degree of wound healing at the line of anastomosis was graded on a scale of 1 to 5: Grade I: Fibrinopurulent exudate, Grade II: Granulation tissue less than 25%, Grade III: Granulation tissue between 25–75%, Grade IV: Granulation tissue more than 75% or collagen fibers less than 25%, Grade V: collagen fibers more than 25%.

### Measuring Hydroxyproline level

Two third of 2-cm segment of the colon including the line of anastomosis was washed with distilled water, dried with blotter, sectioned into little pieces during tissue homogenization and kept frozen at -80°C until the day of the test. Hydroxyproline level was determined by modifying the method described by Woessner [10]. During the procedure, OH-P standard, chloramin-T, P-dimethyl amino benzaldehyde, Perchloric acid, Isopropanol, Na asetat-3 H2O, Na citrate 5,5 H2O, 12 N HCL, 1 mM HCL chemicals were used. Reagents were added onto the sample and blank in order, vortexed and incubated in water bath at 60°C for 25 minutes. Optical density was measured at 558 nm and the concentration of hydroxyproline was calculated by comparing it with the blank. Results are expressed as mg/g dry tissue.

### Statistical analyses

Data obtained from the study group were expressed as mean ± standard deviation. Differences in parameters among groups were examined using One-way analysis of variance (ANOVA) and post-hoc analyses with tukey B and Scheefe tests. Probability (p) value <0.05 was accepted significant.

## Results

No complications including intra-abdominal hemorrhage, infection, anastomosis leak, fistula, or abscess were observed. There were no mortalities during the study.

**Figure 1:**
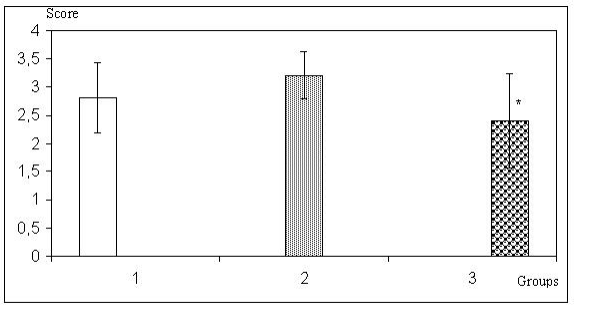
Adhesion grading of the groups * p < 0.05, Group 3 versus Group 2.

Intra-abdominal adhesion score of Group 3 was significantly lower than remained groups (p < 0.05). Figure 1 reflects these data.

**Figure 2:**
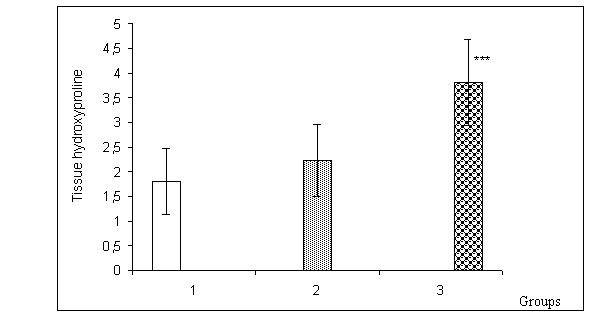
Tissue hydroxyproline levels in groups (mg/g dry tissue). *** p < 0.001, Group 3 versus Group 1 and group 2.

Tissue hydroxyproline levels are presented in Figure 2. Levels in Group 3 were significantly higher compared to the Groups 1 and 2 (p < 0.001).

**Figure 3:**
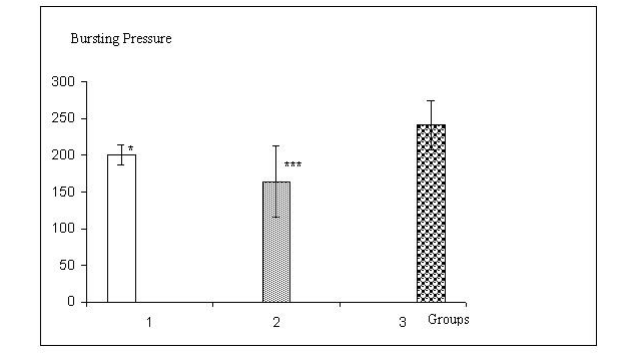
Bursting pressures in groups(mmHg) * p < 0.05, Group 1 versus Group 3. *** p < 0.001 Group 2 versus Group 3.

Mean anastomosis bursting pressures were 200 mmHg, 164 mmHg and 240 mmHg in Groups 1, 2 and 3, respectively. Group comparison showed that anastomosis bursting pressure in Group 3 was significantly higher than the other groups (p < 0.05 between groups 1 and 3; p < 0.001 between groups 2 and 3). Results are presented in Figure 3.

**Figure 4:**
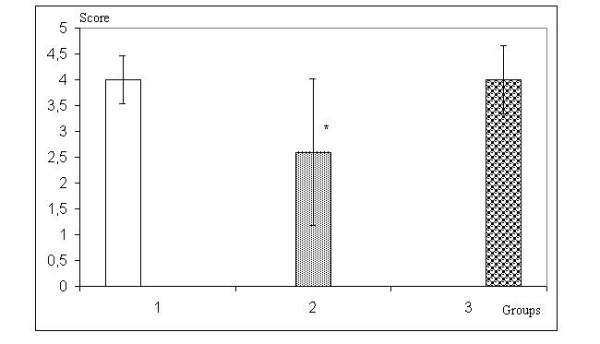
Healing score of the intestinal tissue * p < 0.05, Group 2 versus Group 3.

On histopathological examination healing score of the intestinal tissue was significantly higher in Group 3 than in Group 2 (p < 0.05). Results are presented in Figure 4.

## Discussion

In the present study, we investigated the effects of intra-peritoneally administrated Bosentan on wound healing following resection and anastomosis of a segment of ischemia-induced colon. Bosentan is an endothelin receptor antagonist. When Bosentan administered intraperitoneally, anastomosis bursting pressure, tissue hydroxyproline level, and histopathological anastomosis healing score were higher additionally intra-abdominal adhesion scores were also lower. These results suggest that bosentan increases tissue blood flow by inhibiting the vasoconstriction effect of endothelins.

Jhonson et al [11] have demonstrated that mesenteric vessels have ET-A and ET-B receptors. Moreover bolus administration of ET-1, ET-3, proendothelin 1 or proendothelin 3 into the mesenteric circulation resulted in marked decrease in blood flow. This suggests that mesenteric vascular bed is sensitive to endothelin peptides [12]. It has been shown in an in-vivo study that Sarafotoxin 6C, an ET-B receptor agonist, caused dose-dependent, long-term vasoconstruction in the mesenteric vascular area [13]. Infusion of ET-1 into the superior mesenteric artery increases mucosal permeability, causes accumulation of polimorphonuclear cell (PMN) and mucosal dysfunction [14]. ET-1 is known to play an important role of in the pathogenesis of gastrointestinal mucosal damage whereas there is little information on the effects of ET-3 on the alterations on intestinal inflammatory responce. Small intestines are the major source of ET-3 production [15]. ET-3 decreases superior mesenteric artery (SMA) blood flow significantly and plays important roles in intestinal mucosal dysfunction and tissue PMN infiltration [16]. In the present study tissue endothelin levels were not assayed but in experimental strangulated small bowel obstructions, the release and concentration of ET-1 has been shown to increased in the venous blood of strangulated intestinal loop. In the same study Endothelin-1 has been shown to increase tissue vascular resistance in the intestinal mucosa of the strangulated specimen significantly following its induction in strangulated obstruction. It has been shown that animals that received Bosentan treatment had significantly lower mucosal vascular resistance compared to untreated animals [6].

Bosentan is a non-peptide receptor antagonist of both ET-A and ET-B receptors. Bosentan can be administered orally or parenterally. Its half life is 5.4 hours. In this study Bosentan was administered intraperitoneally. We concluded that Bosentan was not effected only locally. Bosentan is used in the treatment of pulmonary hypertension. There are ongoing research on the effects of Bosentan in heart failure, hypertension, subarachnoidal hemorrhage, acute and chronic renal failure, migraine, and coronary artery disease [17]. To the best of our knowledge there are no studies on the effects of Bosentan on wound healing.

Wound healing in the gastrointestinal system involves related processes such as hemostasis and inflammation, proliferation-fibroplasia and maturation and remodeling. Delay or problem in any of these stages results with a delay in healing [18]. There are numerous local and systemic factors that affect anastomotic healing. The most important local factor is the perfusion and oxygenation of the site of anastomosis. To achieve safe anastomosis, intestinal blood flow should exceed 30% [19-21]. Becaplermin, a platelet derived growth factor has been used locally on normal and ischemic colon anastomosis and eliminated the unfavorable effects of ischemia on the colon anastomosis [22]. We believe that Bosentan decreased the adverse effects of ischemia on the mucosa and submucosa during the initial stage of wound healing, thus positively contributed to wound healing parameters.

Adhesions are encountered after many intra-abdominal surgical procedures. Tissue ischemia is believed to be the most important etiological factor in the adhesion formation because vascular bridges are formed between the ischemic and adjacent healthy organs [7]. The fact that adhesion scores in Bosentan-administered group was lower than the other groups led us to believe that this was due to increase of blood flow on splanchnic area.

As a result, Bosentan decreased the adhesion formation and increased anastomotic healing following resection and anastomosis for strangulated colonic obstruction. We believe the findings of this study should be warrented by large numbered experimental and prospective clinical studies.
